# Supplementary material for: Quiescence modulates age-related changes in the functional capacity of highly proliferative canine lung mesenchymal stromal cell populations
Source: PLoS One. 2025 Jul 23;20(7):e0319723. doi: 10.1371/journal.pone.0319723 (PMC12286334; doi:10.1371/journal.pone.0319723)
Supplement: Attachment — Submitted filename: comments.pdf [file pone.0319723.s003.pdf]

This study investigates whether age-related declines in canine lung mesenchymal stromal cells (LMSCs) are driven by senescence or a quiescent state. The authors do a good job of aligning their findings with a small but growing body of literature that challenges the traditional assumption that senescence is always the primary mechanism of proliferative decline in aging cells.

Comments:

1. Clarify how normality and equal variance assumptions were tested. If multiple t-tests were used, specify any methods (e.g., Bonferroni) to correct for multiple comparisons.
2. Provide numeric values (mean ± SD or SEM) alongside p-values for the main figures.
3. Briefly discuss known regulatory pathways of quiescence and how they might drive LMSC behavior with age.
4. Clarify if any sex- or breed-related differences appear in the data. While the study uses predicted lifespan to help account for breed size, acknowledging or ruling out sex/breed differences would strengthen the analysis.
