# Supplementary material for: Quiescence modulates age-related changes in the functional capacity of highly proliferative canine lung mesenchymal stromal cell populations
Source: PLoS One. 2025 Jul 23;20(7):e0319723. doi: 10.1371/journal.pone.0319723 (PMC12286334; doi:10.1371/journal.pone.0319723)
Supplement: Attachment — Submitted filename: Aygapong et al response to reviewers.docx [file pone.0319723.s005.docx]

Dear Dr. Haque and Reviewers,

Thank you so much for your thoughtful review of this manuscript. We are very grateful for your comments and I believe that they will strengthen this study. Below are our responses to the comments from reviewer 1 (in green):

Reviewer #1: This study investigates whether age-related declines in canine lung mesenchymal stromal cells (LMSCs) are driven by senescence or a quiescent state. The authors do a good job of aligning their findings with a small but growing body of literature that challenges the traditional assumption that senescence is always the primary mechanism of proliferative decline in aging cells.

Comments:
1. Clarify how normality and equal variance assumptions were tested. If multiple t-tests were used, specify any methods (e.g., Bonferroni) to correct for multiple comparisons.

Normality was confirmed using the Shapiro-Wilk test. Equal variance assumptions were confirmed using F-tests. Please see lines 259-262 in the methods.

2. Provide numeric values (mean ± SD or SEM) alongside p-values for the main figures.

Done (please see relevant figure legends in the manuscript).

3. Briefly discuss known regulatory pathways of quiescence and how they might drive LMSC behavior with age.

Done. Please see lines 546-554 in the discussion.

4. Clarify if any sex- or breed-related differences appear in the data. While the study uses predicted lifespan to help account for breed size, acknowledging or ruling out sex/breed differences would strengthen the analysis.

Done. Please see lines 480-48 in the discussion.
