# Supplementary material for: Quiescence modulates age-related changes in the functional capacity of highly proliferative canine lung mesenchymal stromal cell populations
Source: PLoS One. 2025 Jul 23;20(7):e0319723. doi: 10.1371/journal.pone.0319723 (PMC12286334; doi:10.1371/journal.pone.0319723)
Supplement: Attachment — Submitted filename: Aygapong et al 2nd response to reviewers.docx [file pone.0319723.s006.docx]

Dear Dr. Haque and Reviewers,

Thank you so much for your thoughtful review of this manuscript. Below are our responses to the comments from reviewer 3 (in green):

Reviewer #3: The study investigates the senescence of mesenchymal stromal cells isolated from young and old donors in a dog model. The authors express the results considering cellular functions and proliferation capacity clearly and logically.

1. Why was an animal model used in this study? Would it have been possible to conduct a clinical research? Can the results be applied clinically?

An animal model was used in this study to facilitate ease of sample collection, to ensure access to donor age, and to explore MSC function across diverse animal species. A clinical research study would either require lung biopsies from patients, or a post-mortem sample collection system. Given the similarities between companion dog and human health-spans (see line 84-87 of the manuscript), we believe that the results from our study can be applied clinically.

2. Can you clarify why mesenchymal stromal cells were derived from lung? Adipose derived mesenchymal stem cells would be easier to obtain.

We were specifically interested in understanding how MSC function changed with donor age when isolated from a low turnover tissue system such as the lung that has clinically relevant increased pathology and chronic disease with age (see lines 37-41 in manuscript). In addition, in this study design, MSCs derived from post-mortem lung tissue are as easy to obtain as those derived from adipose tissue.

3. There are 16 authors in this study. What was the contribution of each author to this work?

The work in this study was performed by myself and teams of undergraduate research students (for a total of 16 researchers). Each team of students designed and optimized the assay that they were working on, analyzed the data, and contributed to writing the final manuscript. Because they were undergraduate students, each team worked on only one o the assays in the study, but all the authors listed contributed significantly to the study design, implementation, data interpretation, and manuscript writing.
